# Sedentary Lifestyle and Estimated Fracture Risk in the Elderly: A Cross-Sectional Study

**DOI:** 10.7759/cureus.104326

**Published:** 2026-02-26

**Authors:** Tommy A Masal, Erica Kholinne

**Affiliations:** 1 Surgery, Faculty of Medicine, Universitas Trisakti, Jakarta, IDN; 2 Orthopedics and Traumatology, Gatam Institute, Eka Hospital, Tangerang, IDN

**Keywords:** elderly, fracture risk, frax, physical activity, sedentary lifestyle

## Abstract

Background

Fractures are a major public health concern among the elderly and contribute substantially to morbidity and disability. Sedentary behavior has been proposed as a modifiable risk factor for fractures; however, evidence regarding its association with fracture risk remains inconsistent. This study aimed to evaluate the relationship between a sedentary lifestyle and fracture risk among elderly individuals in a nursing home setting.

Methods

This cross-sectional study was conducted in October 2025 at Tresna Werdha Budi Mulia 2 Social Home, Jakarta. A total of 93 participants aged ≥60 years were recruited using consecutive sampling. Physical activity levels were assessed using the International Physical Activity Questionnaire (IPAQ), and fracture risk was evaluated using the Fracture Risk Assessment Tool (FRAX) without bone mineral density. Fracture risk was categorized as low (<5%) or moderate-to-high (≥5.0-7.5%). Statistical analysis was performed using SPSS v23, and the Fisher-Freeman-Halton exact test was used to assess the association between physical activity level and fracture risk.

Results

The study included 93 participants, consisting of 50 males (53.8%) and 43 females (46.2%), aged between 60 and 91 years, with a mean age of 69.7 ± 7.5 years. Most participants were classified as sedentary (n=62, 66.7%) and had a low fracture risk (n= 75, 80.6%). No statistically significant association was found between sedentary lifestyle and fracture risk (p = 0.232).

Conclusion

A sedentary lifestyle was not significantly associated with fracture risk among elderly nursing home residents. These findings indicate that fracture risk is likely influenced by multiple interacting factors beyond physical activity alone. Further longitudinal studies using objective physical activity measurements are needed to clarify this relationship.

## Introduction

In Indonesia, older adults are defined as individuals aged 60 years or older. Population aging is occurring worldwide, with a number expected to rise to 1.5 billion by 2050 [1]. Projections from the Central Statistics Agency (Badan Pusat Statistik), Indonesia, in 2024 indicate that older adults will comprise 12.00% of the total population, signifying the country’s transition into an aging society, defined by an elderly population proportion exceeding 10% [2].

A fracture is defined as a partial or complete disruption of bone continuity. Osteoporotic fractures pose a major global health burden, with an estimated lifetime risk of one in three women and one in five men [3]. Fractures in older adults commonly involve the wrist, upper extremity, spine, foot, ankle, and hip, with hip fractures representing one of the most clinically significant injuries. Hip fractures account for approximately 18.2% of all fractures and are associated with high morbidity and mortality rates [4]. In Indonesia, the most frequently reported fracture types among older adults include vertebral (25%), hip (24%), wrist (13.6%), and proximal humeral fractures (7.2%). Data from the 2018 Basic Health Research (RISKESDAS) reported an overall fracture prevalence of 5.5% nationwide [5].

Fracture risk in older adults arises from both intrinsic and extrinsic factors. Most fractures are related to falls, often resulting from impaired balance and mobility. Intrinsic contributors include visual impairment, vestibular dysfunction, and neuromusculoskeletal disorders [6]. Extrinsic factors, such as unsafe environments, poor lighting, and slippery surfaces, further increase the fall risk [7,8]. A sedentary lifestyle refers to waking activities performed in a sitting or reclining posture, which involves minimal energy expenditure, defined as ≤1.5 metabolic equivalents (METs) [9]. In Indonesia, approximately 21.1% of older adults engage in sedentary behavior, indicating that nearly one in five elderly individuals has low levels of physical activity [10]. Fractures represent a major public health concern and are a leading cause of disability among the elderly, largely driven by age-related changes in bone remodeling, alterations in the bone microenvironment, and additional modifiable risk factors [4,11]. 

This study aimed to examine the association between sedentary lifestyle, defined as low physical activity measured by the International Physical Activity Questionnaire (IPAQ), and estimated fracture risk assessed using the Fracture Risk Assessment Tool (FRAX) without bone mineral density [12-14]. The outcome of interest was the calculated 10-year probability of major osteoporotic fracture rather than actual fracture occurrence.

## Materials and methods

This study employed an observational cross-sectional design. The research was conducted at Tresna Werdha Budi Mulia 2 Social Home, located in West Cengkareng, Jakarta, Indonesia, in October 2025. The study population comprised elderly residents of the social home. Participants were enrolled using consecutive sampling, in which all eligible individuals meeting the inclusion criteria during the study period were included. Inclusion criteria were age ≥60 years and adequate ability to communicate. Exclusion criteria comprised a history of fracture within the previous six months, long-term systemic glucocorticoid use for more than three months, and unwillingness to participate. The study received ethical approval from the Research Ethics Committee of the Faculty of Medicine, Universitas Trisakti (approval number: 001/KER/FK/10/2025). All participants received an explanation of the study procedures and provided written informed consent before enrollment.

This study used instruments to assess physical activity and fracture risk. Physical activity was measured using the seven-item International Physical Activity Questionnaire (IPAQ) short form, Indonesian version, which evaluates activity over the past seven days. The questionnaire was interviewer-administered by trained research personnel to minimize misinterpretation and recall bias, and standardized prompts were used when necessary to ensure consistent understanding of activity intensity. Physical activity levels were assessed based on participants’ total weekly energy expenditure, expressed in metabolic equivalent task (MET)-minutes per week. In accordance with established criteria, participants were classified into three categories: low physical activity (<600 MET-minutes/week), moderate physical activity (≥600 MET-minutes/week), and high physical activity (≥3,000 MET-minutes/week). This categorization reflects increasing levels of physical activity intensity and duration, with higher MET-minute values indicating greater overall energy expenditure [11-13].

Fracture risk was evaluated using the Fracture Risk Assessment Tool (FRAX). The country-specific FRAX model for Indonesia was used to estimate fracture probability. FRAX provides an estimate of the 10-year probability of major osteoporotic fracture, including hip, clinical spine, forearm, and humerus fractures. Data were collected for 12 variables, including age, sex, body weight, height, previous fracture, parental history of hip fracture, rheumatoid arthritis, secondary osteoporosis, smoking status, alcohol consumption, and glucocorticoid use [14]. Bone mineral density (BMD) was not included in the FRAX calculation. Participants were classified as having low fracture risk if the ten-year probability of an osteoporotic fracture was <5%, and as having moderate-to-high fracture risk if the probability was ≥5.0% to ≥7.5% [3,11]. Study procedures began with participant screening based on predefined inclusion and exclusion criteria, conducted with assistance from nursing home staff. All collected data were reviewed for completeness at the time of collection, and participants with incomplete key variables required for IPAQ scoring or FRAX calculation were excluded from the final analysis. Data entry was cross-checked to maintain accuracy and consistency.

Statistical analysis

All statistical analyses were performed using the Statistical Package for the Social Sciences (SPSS, v 23. IBM Corp., Armonk, NY, US). Univariate analysis was conducted to summarize each variable, with percentage distributions calculated for age, sex, fracture risk, and physical activity level. Bivariate analysis was initially performed using the chi-square test; however, because the assumptions related to expected cell counts were not satisfied, the Fisher-Freeman-Halton exact test was used. Statistical significance was defined as a p-value of <0.05.

## Results

**Figure 1:**
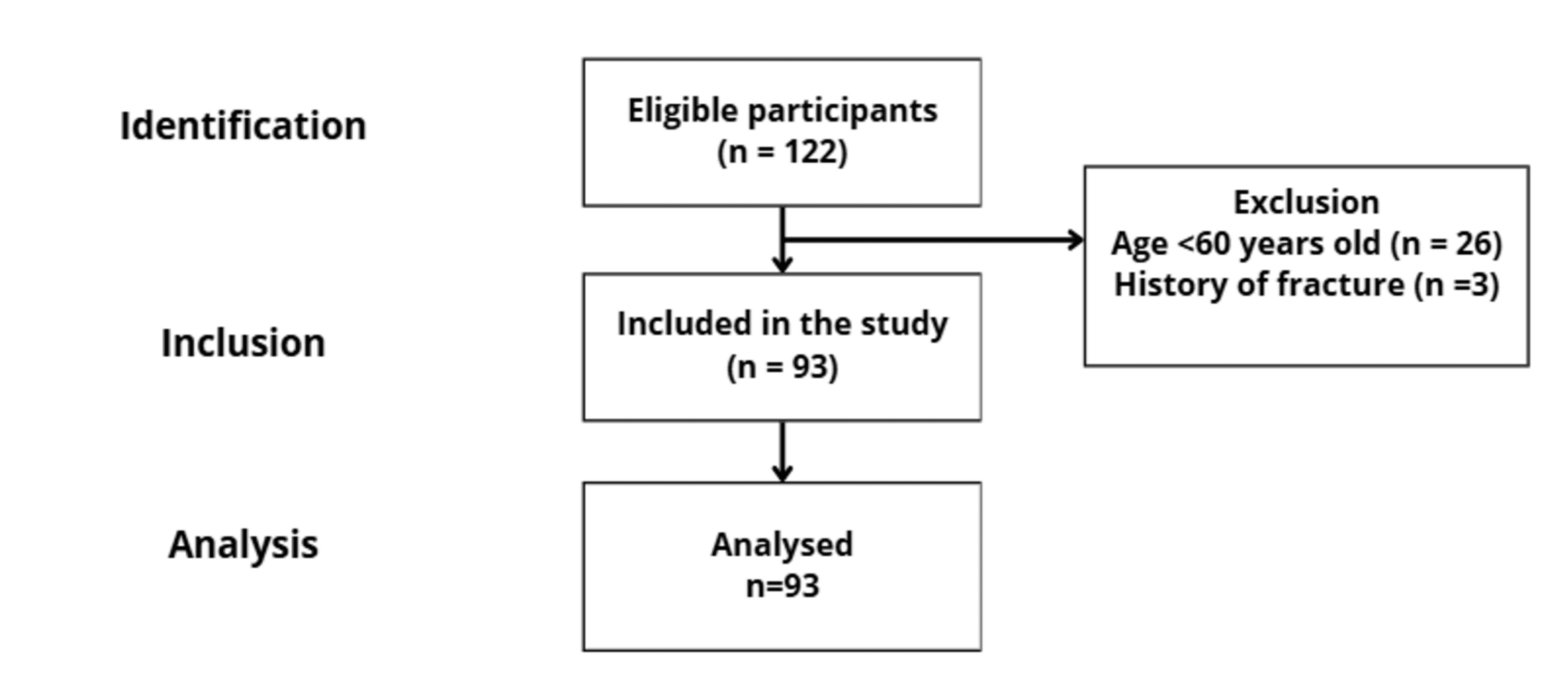
Flowchart of participant selection in the study

A total of 122 individuals were assessed for eligibility, while 93 participants were included in the final analysis (Figure 1).

This study comprises 50 (53.8%) males and 43 (46.2%) females, aged 60 to 91 years, with a mean age of 69.7 ± 7.5 years old. Seventy-five (80.6%) participants were classified as having a low fracture risk based on the FRAX questionnaire, and 62 (66.7%) participants were in the sedentary group based on the IPAQ questionnaire. Detailed participant characteristics are presented in Table 1.

**Table 1:** Demographic characteristics of the participants IPAQ: International Physical Activity Questionnaire; FRAX: Fracture Risk Assessment Tool

Characteristics	N=93	Percentage (%)
Gender		
Male	50	53.80%
Female	43	46.20%
FRAX Questionnaire		
Low fracture risk	75	80.60%
Moderate-high fracture risk	18	19.40%
IPAQ Questionnaire		
Sedentary	62	66.70%
Moderate activity	26	27.90%
High activity	5	5.40%

Table 2 summarizes the distribution of fracture risk across physical activity levels as measured by the IPAQ. The majority of participants reported low physical activity (62; 66.7%), while 26 (28.0%) reported moderate activity and 5 (5.4%) reported high activity levels. Across activity categories, most participants were classified as having a low fracture risk. Statistical analysis revealed no significant association between physical activity level and fracture risk (p = 0.232).

**Table 2:** Relationship between physical activity level and fracture risk in the elderly ‡: Fisher-Freeman-Halton Analysis (p = 0.232) METs: metabolic equivalents; IPAQ: International Physical Activity Questionnaire; FRAX: Fracture Risk Assessment Tool

IPAQ Score	FRAX				Total	p-value
	Low risk (<5%)	Percentage (%)	Moderate-high risk (≥5.0)	Percentage (%)		
Light activity/sedentary <600 METs-minutes/week	50	53.8%	12	12.9%	62 (66.7%)	
Moderate activity >600 METs-minutes/week	21	22.6%	5	5.4%	26 (28%)	0.232
High activity >3000 METs-minutes/week	4	4.3%	1	1.1%	5 (5.4%)	

## Discussion

In this study, the majority of respondents were found to have low levels of physical activity (66.7%). This proportion is notably higher than that reported by Setiati et al., who found that 21.1% of elderly individuals in Indonesia had low physical activity levels [10]. The discrepancy may be attributable to differences in study settings, as data in the present study were collected from a nursing home environment where opportunities for physical activity are inherently limited. Furthermore, physical activity levels were assessed using self-reported questionnaires without objective verification, which may have introduced reporting bias and contributed to the high prevalence of low physical activity observed [12].

The findings of the present study indicate that most participants were categorized as having a low fracture risk, with 75 individuals (80.6%) falling into this group. A similar study conducted by Mustasmir et al. reported that all older adults assessed using the FRAX tool were classified as having a low fracture risk [15]. This pattern may be partly attributable to the generally low levels of physical activity observed among the study population. Evidence from Van Gameren et al. suggests that high-intensity physical activity is associated with a greater risk of fall-related fractures in older adults compared with low-intensity activity [11].

The results of Fisher’s exact test showed no statistically significant association between sedentary behavior and fracture risk among older adults (p = 0.232). This lack of association may be influenced by unmeasured factors in the current study, including the absence of objective physical activity assessment using accelerometer-based devices. Similar methodological considerations were noted in the study by Giné-Garriga et al., who evaluated physical activity and sedentary behavior using an ActiGraph accelerometer worn continuously for seven days [16]. Although accelerometer-based assessments provide more objective and precise measurements, self-reported questionnaires, while more feasible and cost-effective, may lead to overestimation or underestimation of actual physical activity levels [17].

There are several differing opinions on whether a sedentary lifestyle is linked to an increased risk of fracture. Studies indicate a correlation between a sedentary lifestyle and frailty, which can lead to factors that increase the risk of falls and fractures, such as decreased physical function associated with postural instability and poor balance. Additionally, frailty is also linked to impaired mobility [8]. Therefore, in this study, fracture risk assessment primarily focused on using the FRAX tool, which directly evaluates the risk of major osteoporotic fractures. However, in this study, most participants with a sedentary lifestyle had a low risk of fracture, with only a few having a high risk of fracture.

The findings of the present study are consistent with those reported by Guðmundsdóttir et al., who similarly found no evidence of an association between sedentary behavior and fracture occurrence. Their study highlighted the potential influence of several confounding factors that may affect fracture risk, including genetic predisposition, hormonal changes, socioeconomic status, body composition, and variations in medication use [18]. This shows that physical activity is not an independent factor that determines the risk of fracture.

The results of this study align with a study by Gameren MV et al., which did not find a significant link between fractures and physical activity. The authors even argued that high-intensity physical activity might increase the risk of falls and fractures, in contrast to low-intensity activity [11]. Although this study mentions that high-intensity physical activity can increase bone density and potentially reduce fall risk, these two viewpoints show that physical activity alone does not determine fracture risk [8,11].

This study has several limitations. First, the study population was limited to residents of a single nursing home, which may restrict the generalizability of the results to the wider older adult population. Second, physical activity was not assessed using objective instruments, such as accelerometers, which may have led to less precise measurement. Third, the unequal distribution of fracture risk categories resulted in limited representation of individuals with a moderate-to-high fracture risk. Fourth, the fracture risk was estimated using the FRAX tool without inclusion of bone mineral density (BMD), which may underestimate the fracture probability in some individuals and influence risk classification. In addition, this study evaluated the estimated fracture risk using a prediction model rather than actual fracture events, which may limit direct clinical interpretation of the findings. Furthermore, the definition of elderly as age ≥60 years followed Indonesian national standards and may differ from international definitions, potentially affecting comparability across populations. Lastly, the cross-sectional design prevents causal inference.

## Conclusions

In conclusion, this study found no significant association between a sedentary lifestyle and fracture risk among elderly nursing home residents. These findings suggest that sedentary behavior alone may not independently determine fracture risk, which is likely influenced by multiple interacting factors. Further studies using objective physical activity measurements and longitudinal designs are needed to clarify this relationship.
